# Examining the Relationship between Sugar Content, Packaging Features, and Food Claims of Breakfast Cereals

**DOI:** 10.3390/nu13061841

**Published:** 2021-05-28

**Authors:** Marília Prada, Magda Saraiva, Claúdia Viegas, Bernardo P. Cavalheiro, Margarida Vaz Garrido

**Affiliations:** 1 Department of Social and Organizational Psychology, Iscte-Instituto Universitário de Lisboa, CIS_Iscte, Av. das Forças Armadas, Office AA110, 1649-026 Lisboa, Portugal; magda.saraiva@iscte-iul.pt (M.S.); Bernardo_Cavalheiro@iscte-iul.pt (B.P.C.); margarida.garrido@iscte-iul.pt (M.V.G.); 2 Escola Superior de Tecnologia da Saúde de Lisboa, Instituto Politécnico de Lisboa, Av. D. João II, Lote 4.69.0.1, Parque das Nações, 1990-096 Lisboa, Portugal; claudia.viegas@estesl.ipl.pt; 3 Centre for Tourism Research, Development and Innovation-Pòlo do Estoril, Avenida Condes de Barcelona, n.° 808, 2769-510 Estoril, Portugal

**Keywords:** sugar, claims, labeling, nutritional profile, breakfast cereals

## Abstract

Excessive free-sugar intake has become highly prevalent in numerous countries, and Portugal is not the exception. One product category that contributes to the daily intake of free sugars is breakfast cereals. In the current work, we identified 289 exemplars from two major retailers in Portugal and collected information on their nutritional profile (e.g., sugar, salt, fiber per 100 g), price, packaging features, type of food claims present (e.g., statements about the composition, sensory features, the origin of the product), and ingredients list. Overall, the sugar content of breakfast cereals was high (*Mean* = 19.9 g), and less than 10% of the products complied with the current national guidelines (i.e., 5 g of sugar per 100 g of product). Sugar (or other sugar sources) was listed in the top three ingredients for over 85% of the products. On average, each product included about four claims (*Mean* = 3.9), and sugar content was lower when the claims were related to the product composition. Critically, the sugar content was particularly high for children-oriented products (*Mean* = 26.4 g). Correlation analysis showed that breakfast cereals with higher sugar content also were cheaper and had lower quantities of fiber, proteins, and salt. Our findings suggest the need to implement strategies to reduce sugar in this product category (e.g., incentivize manufacturers to reformulate products). Also, our results may inform strategies aimed at promoting consumers’ awareness about the sugar content in breakfast cereals and other processed foods, facilitating healthier decision-making.

## 1. Introduction

The World Health Organization (WHO) [1] estimated that 650 million people worldwide are obese. In Portugal, the Health at a Glance report [2] indicates that 67.6% of the population over 15 years of age is overweight or obese. Excessive sugar intake—particularly free sugars (i.e., all mono- and disaccharides added to foods or beverages by the manufacturer, cook, or consumer, as well as all the sugars that are naturally present in honey, syrups, fruit juices, and fruit concentrates [3,4])—is strongly associated with obesity. Considering the adverse outcomes of excessive sugar intake, the WHO [4] recommends that both children and adults should limit the consumption of free sugars to less than 10% (ideally 5%) of total energy consumption. In Portugal, although the estimated average intake of free sugars is 7.5% of the total daily energy consumption (i.e., 35 g/day [5]), 24.3% of the population exceeds the recommended 10%. This problem is even more worrying in adolescents (48.7%) and children (40.7% [5]).

The subgroups of foods that most contribute to the daily intake of free sugars include cakes, soft drinks, cookies, breakfast cereals, and nectars, among others [5]. Breakfast cereals, in particular, are ultra-processed food products [6] that typically contain high amounts of sugar, energy, sodium content, and saturated fat, particularly those targeting children [7,8,9,10,11]. For example, Fayet-Moore et al. [12] found that individuals who consume breakfast cereal had a higher intake of total sugars than non-consumers of these products. According to a recent report, breakfast cereals are among the top 20 food groups in terms of sales values in Portugal [13]. Breakfast cereal consumption is particularly prevalent among younger Portuguese individuals (e.g., children and adolescents consume more than double the average for the overall population [5]). Moreover, those who consume breakfast cereals seem to exceed the usual serving size of 30 g (e.g., adolescents ingest on average 49.4 g [5]). Importantly, breakfast cereals are highly heterogeneous in terms of their nutritional quality [8,14,15]. For instance, a recent study of the nutritional characteristics of a sample of 163 breakfast cereals available in the Portuguese market showed that sugar content varied between 0.7 and 48 g (per 100 g of product), and only 15% complied with the current national guidelines for the maximum amount of sugar (i.e., 5 g per 100 g of product) [16]. For this reason, consumers must be able to distinguish between less healthy and healthy food alternatives, namely those with lower sugar content. This decision can often be shaped by the information provided on the packaging.

Packages are designed with different visual and informational attributes that may influence the processing of information regarding the product (see [17,18,19] for reviews). The visual attributes of food packaging are all the elements that set up the product design (e.g., layout, color combination and contrast, photography or illustrations, size, and shape of packaging). For instance, cartoon characters are a feature of food packaging known to capture the attention of consumers, particularly children, promoting the desire for the product [20]. The informational elements might include packaging technology (e.g., “made from recycled material”), but more frequently, they refer to information about the product itself, namely nutritional verbal or numeric information, such as food labels [18,19]. Nutritional information in food products is important to allow the consumer to make informed and healthier choices [21,22]. In the European Union (EU), for example, nutrition labeling is required for all prepackaged food, and the Regulation (EU) No 1169/2011 [23] defined a list of mandatory information to be presented on food packages (e.g., list of ingredients, date of minimum durability, nutrition declaration). All prepackaged food has a Nutrition Facts Panel (NFP) that details the key nutrients for the product [24]. However, at the time of purchase, consumers usually do not have time to attend to all the information presented in the NFPs [25]. For this reason, simpler ways to present the nutritional content (and other characteristics of the products) have emerged, resulting in a variety of front-of-package (FoP) nutrition labels that are used worldwide (see [26] for an overview). These FoP labels include, for example, nutrient-specific labels that report simplified information about a specific nutrient (e.g., sugar and fat content represented in a nutritional traffic light system) or the overall healthfulness of the product (e.g., health star ratings; see [27,28] for reviews).

The credibility of the product (and its manufacturer) is often inferred by the information presented in food packaging (for a review on credence cues, see [29]). Besides the brand, these credence cues [29] include: (a) statements about the beneficial nutritional properties of the product (e.g., low sugar) and/or how it may impact consumers’ health (e.g., “calcium may reduce the risk of osteoporosis”; for a review, see [28]); (b) attributes related to the food origin, production method, and ethical or environmental concerns (e.g., country of origin, organic farming, fair trade, animal welfare); and (c) descriptive food names (e.g., names that elicit sensory experiences or appeal to memories and tradition). Importantly, based on these cues, consumers infer product characteristics and generate expectations that may be unsubstantiated. For instance, research has shown that consumers perceive products with health (for a review, see [30]) and organic claims as more healthful (e.g., [31,32]) than products without those claims. Moreover, these claims often lead to positive inferences about unrelated attributes (e.g., lower caloric content), an effect known as the health halo [33].

Concerning the case of breakfast cereals, studies conducted in different countries have examined the nutritional quality of the products according to the labeling presented in the packages. However, the observed results seem to be mixed. Some studies found that the differences in healthfulness of breakfast cereals with (vs. without) nutritional and health claims were limited [34] or non-significant [35]. Other studies found significant differences but in the opposite direction. For example, Devi et al. [8] found that nutritional claims were more frequently found in healthier options, whereas Nieto et al. [9] found that claims were more frequently displayed in breakfast cereals with higher energy, sodium, and sugar content.

The main goal of the current study was to characterize the sugar content of a large sample of products (289 breakfast cereals) gathered from the Portuguese market. We describe sugar content along with other nutrients (e.g., fiber, salt) and categorize sugar levels according to the nutritional traffic light system. Besides the focus on the nutritional profile, we also categorized the features of the products’ packaging. Specifically, we describe the types (and frequency) of the FoP claims as well as other relevant packaging/product information (e.g., child-oriented design, price). Moreover, we examined how these informational features were associated with the sugar content of the analyzed products.

## 2. Method

### 2.1. Products Description and Data Collection

This study includes data regarding 289 breakfast cereals from 28 brands (74.4% of the products were name-brands such as Nestlé, Kellogg’s, or Alpen, and the remaining were own-label brands). The data was gathered between March and June 2019 using the websites of two of the top retailers in the Portuguese market. These retailers hold the highest market share in Portugal (i.e., Continente–Sonae holds the first position and Auchan the 3rd; Pingo Doce–Jerónimo Martins was not included, as their website does not present products’ full nutritional information), also leading in food retail e-commerce [36,37]. Table 1 summarizes the information collected. The search was conducted using a variety of keywords associated with this product category (e.g., breakfast cereals, cereals, granola, muesli, etc.). All information was compared between retailers’ websites and, if necessary, (e.g., absence or inconsistencies of online information) with the brands’ website or physical stores. Duplicates were removed and, whenever different sizes of the same product were available (e.g., regular and family size), the regular size was selected. Package quantity for breakfast cereals varied between 175 g and 1000 g (*M* = 393.27, *SD* = 115.96). Whenever the product was available in both retailers, we calculated the mean price (per kg), which varied between 1.97€ and 35.63€ (*M* = 9.83, *SD* = 6.16).

**Figure 1:**
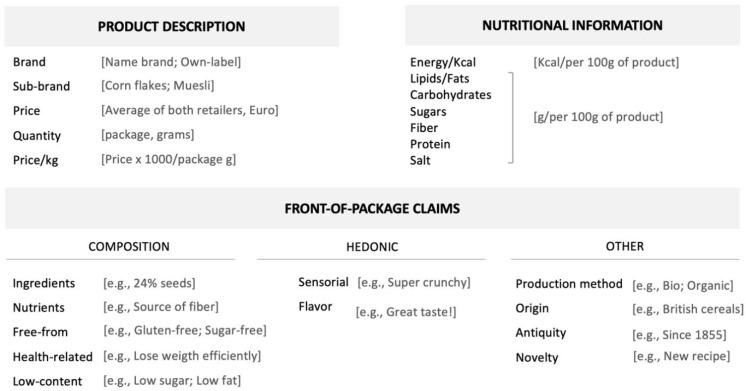
Categories (and examples) of information gathered for each product.

For each product, we gathered images of the front and the back of the package. Besides the information described in Figure 1, we also categorized other packaging elements, such as the presence of children-oriented design (e.g., cartoon characters, cartoonish fonts, see also [11,20]) and traffic light labeling in the front of the package, as well as the list of ingredients.

### 2.2. Data Analytical Plan

Data were analyzed with SPSS v29, and an alpha level of 0.05 was used for the inferential analyses. First, we characterize the nutritional information gathered for the set of breakfast cereals. Specifically, in Section 3.1, we present:

- descriptive statistics (mean, standard deviation, median) of energy (Kcal) and nutrients (g) per 100 g of product;
- the categorization of sugar, salt, fat, and saturated fat according to the nutritional traffic light system; and
- the pattern of associations (Pearson’s correlation coefficient) between exemplars’ sugar content with the other nutrients, energy, and price.

Subsequently, in Section 3.2, we characterize the different types of claims presented in the set of breakfast cereals and examine whether the sugar content of the products varies according to the presence of each type of claim (independent samples *t*-test).

In Section 3.3, we present results regarding the ingredients list, namely the top three ingredients of the breakfast cereals analyzed.

Finally, in Section 3.4, we examine if sugar content varied according to packaging features, namely child-oriented design (independent sample *t*-test) and different nutritional traffic light labels (one-way ANOVA, post hoc comparisons with Bonferroni correction).

## 3. Results

### 3.1. Nutrition Information

**Figure 2:**
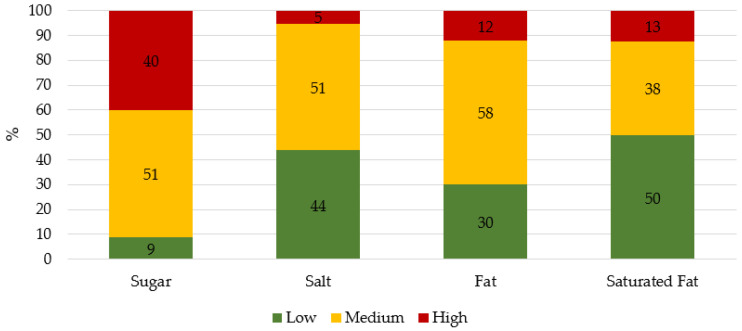
Categorization of sugar, salt, fat, and saturated fat levels according to the nutritional traffic light system. Levels for each nutrient (g/100 g product): sugar (low < 5, medium 5–22.5, high > 22.5); salt (low < 0.3, medium 0.3–1.5, high > 1.5); fat (low < 3, medium 3–17.5, high > 17.5) and saturated fat (low < 1.5, medium 1.5–5, high > 5) [39,40].

We collected information about the energy and nutrients present in 100 g of product. As shown in Table 1, breakfast cereals contained between 0 and 45.2 g of sugar. On average, one-fifth of the products’ composition was sugar (*M* = 19.9, *SD* = 8.8, *Me* = 20.3). Moreover, only 25 (8.7%) of the products complied with the current national guidelines about sugar content (i.e., less than 5 g of sugar per 100 g of product, Dispatch No 11418, 2017 [38]). This would correspond to being classified as green for sugar content in a nutritional traffic light system. As shown in Figure 2, the nutrient that is most frequently present in high proportion is sugar (40% of products are categorized as high in sugar versus 13% high in saturated fat, followed by 12% high in fat and 5% high in salt content).

We also examined how sugar content was associated with other nutrients, energy (Kcal), and price. Sugar content was negatively correlated with fiber, *r* = −0.31, *p* < 0.001; protein, *r* = −0.38, *p* < 0.001; and salt, *r* = −0.18, *p* = 0.002. That is, breakfast cereals with higher sugar content also had lower quantities of fiber, proteins, and salt. Sugar content was positively associated to total carbohydrates, *r* = 0.30, *p* < 0.001. The correlations between sugar content and total fat, *r* = −0.10, *p* = 0.114; saturated fat, *r* = 0.08, *p* = 0.203; and energy (kcal), *r* = 0.01, *p* = 0.831 were non-significant. The amount of sugar was negatively correlated with price (per kg), *r* = −0.16, *p* = 0.006, revealing that inexpensive breakfast cereals were the ones containing higher amounts of sugar.

### 3.2. Claims

Overall, based on the analysis of the sample of 289 breakfast cereals, we identified a total of 1129 claims (i.e., on average, each product contained 3.9 claims). Most of these claims (*n* = 786, 69.6%) were related to product composition, 140 (12.4%) were related to hedonic properties, and 203 (18.0%) referred to other dimensions. Table 2 summarizes the percentage of products with zero, one, two, or more than three claims of a given type. Composition claims regarding the presence of specific nutrients (e.g., “12 vitamins”) were particularly prevalent. Hedonic claims were less common (e.g., “delicious”). Finally, among the other claims, the most prevalent were the ones related to the production method (e.g., “produced with ingredients from organic agriculture”).

Moreover, we compared sugar content according to the presence (vs. absence) of claims. Products with composition claims contained significantly less sugar (*M* = 19.1, *SD* = 8.8) than products without such claims (*M* = 25.1, *SD* = 6.5), *t*(284) = 4.16, *p* < 0.001, *d* = 8.53, 95%CI [0.37,1.05]. In contrast, sugar content of products with hedonic claims (*M* = 19.0, *SD* = 8.9) did not significantly differ from those without such claims (*M* = 20.4, *SD* = 8.7), *t*(284) = 1.29, *p* = 0.198. Finally, products with other claims (*M* = 18.5, *SD* = 8.4) contained less sugar than the ones without such claims (*M* = 21.0, *SD* = 8.9), *t*(284) = 2.33, *p* = 0.020, *d* = 8.71, 95%CI [0.04,0.51].

### 3.3. Ingredient List

We also collected data from the ingredient list regarding the top three ingredients of the breakfast cereals. To summarize this information (see Table 3), we aggregated similar ingredients, except for sugar-related ingredients.

As shown in Table 3, unsurprisingly, the main ingredient of most products (95.8%) is cereals, which is also prevalent as the second (23.5%) or third (19.7%) ingredients. Only three products (1.0%) included a sugar-related item (honey) as the first ingredient. Noteworthy, sugar (including different types of sugar, such as beet or cane sugar, as well as other sweeteners such as honey or syrups) was the second ingredient in more than half of the products (51%). Sugar-related ingredients were also very prevalent as the third ingredient (34.4%).

### 3.4. Differences in Nutritional Quality According to the Presence of Cartoon Characters and Nutritional Traffic Light

We explored whether the sugar content of products varied according to the presence of cartoon characters (i.e., products oriented to children) or to the presence (and format) of the nutritional traffic light. Indeed, when the packaging included cartoons, the products included more sugar (*M* = 26.4, *SD* = 6.4) than when those elements were absent (*M* = 18.5, *SD* = 8.6), *t*(284) = −6.27, *p* < 0.001, *d* = 8.24, 95%CI [−1.27,−0.65].

Regarding the nutritional traffic light, we compared sugar content for four categories: absent (*M* = 17.4, *SD* = 8.8, *n* = 133), present in a standard format (*M* = 20.7, *SD* = 9.4, *n* = 42), present without the traffic light colors (i.e., summary of main nutritional categories, in black and white, or in the same color of package—*M* = 21.9, *SD* = 7.9, *n* = 77), present without the traffic light colors, and focused on a single nutritional characteristic (e.g., energy/calories per serving size presented in green, blue, or black and white—*M* = 24.6, *SD* = 6.5, *n* = 34), *F*(3282) = 8.97, *p* < 0.001, *n*_p_^2^ = 0.087. Packages without any type of nutritional traffic light contained less sugar than the remaining, *p* ≤ 0.002, except for the standard nutritional traffic light without colors, *p* = 0.172 (multiple comparisons with Bonferroni correction).

## 4. General Discussion

Excessive sugar intake is a major problem, particularly because it is associated with negative health outcomes, such as overweight and obese states [4]. In Portugal, these conditions are very prevalent, and about one-fourth of the adult Portuguese population exceeds the WHO recommendation for sugar intake [5]. In the current study, we focused on a product category (breakfast cereals) that contributes to this high sugar intake. We analyzed all the breakfast cereals available in two major retailers and selected 289 exemplars of this category (e.g., granola, muesli, puffed cereals, cereal flakes), which illustrates the array of products available in the Portuguese market. We collected data on the nutritional profile of these products and coded several features of the product’s packaging (i.e., claims; presence of nutritional traffic light; child-oriented design). The analyses focused on defining the sugar content of the set of products and examining the relationship between sugar content and packaging features.

The breakfast cereals analyzed varied widely in terms of sugar content (i.e., from 0.7 up to 45 g of sugar per 100 g of product). Specifically, while (a few) products did not include sugar at all, in other products, sugar was almost half of the products’ composition. Still, a key finding is that, on average, the sugar content was high, representing one-fifth of the product’s composition. These results are similar to those reported in various studies focusing on the nutritional quality of breakfast cereals available in different countries (e.g., Italy [8], New Zealand [9], and Mexico [34]). Critically, when we categorized the products according to the nutritional traffic light parameters, we observed that sugar was the main nutrient for this product category (e.g., 40% of exemplars categorized as red regarding sugar content vs. 13% of exemplars categorized as red regarding saturated fat content). Indeed, only 8.7% of the analyzed products follow the current recommendations in Portugal (i.e., less than 5 g of sugar per 100 g of product). These results are in line with those reported by Fernandes et al. [16], namely that 89.2% (*n* = 167) of the analyzed breakfast cereals in Portugal did not comply with this recommendation.

Moreover, we also found that sugar content was negatively associated with other nutritional indicators, namely fiber, and protein. This is doubly detrimental, as these products have higher amounts of sugar along with less fiber and protein, which are nutrients that contribute to satiety, glucose control, and weight management [41,42,43]. Salt was also negatively associated with sugar content. This could be explained by the fact that salt is often used as a flavor enhancer [44]. Nevertheless, only 5% of the analyzed products had a high salt content. We did not find significant associations between sugar content and energy or fat content. This might be expected since sugars are a part of total carbohydrates and provide fewer calories than fat. Also, cereals are natural sources of carbohydrates and do not provide significant amounts of fat.

Noteworthy, we also found that, in our sample of breakfast cereals, the lower the price, the higher the sugar content. This result is particularly critical since it is well established in the literature that price is a driver of food choice [45] and is one of the most reported barriers to healthy eating (see for a review [46]). In the current study, we did not assess whether consumers actually consume these less expensive products more frequently than breakfast cereals with lower sugar content. Nevertheless, other studies indicate that if the price of a particular food increases, its consumption decreases and vice versa [47]. Additionally, lowering the price of healthier foods and raising the price of less healthy ones can lead to a change in intended food choices (e.g., [48]). For this reason, future studies should investigate the actual consumption patterns of these products and explore the relationship between price and consumers’ perceptions of other attributes of the product (e.g., healthiness, taste).

The analyses of the ingredients list showed that sugar was present among the top three ingredients for about 85% of the products. In most cases, “sugar” was listed, but variations were also found (e.g., cane sugar; beet sugar; coconut sugar). Moreover, we also found references to other terms related to sugars (e.g., honey, maltodextrin, syrups). This is not surprising, as over 150 types of free sugars have been identified in ingredient lists [49], and consumers often have difficulties in recognizing these types of ingredients as sugar sources (e.g., [50]). Research also showed that individuals often report negative attitudes toward sugar (e.g., [51]), which may explain why breakfast cereals with “fruit sugar” are perceived as more healthful than the same products with “sugar” [52]. Therefore, future studies could investigate whether different terms used to describe sugars influence consumers’ food perception and decision-making, particularly for high-sugar products such as breakfast cereals.

The claims presented in the food packaging were also analyzed. Overall, claims were highly prevalent. Converging with the results reported by Devi et al. [8], on average, each product included around four claims. The results also revealed that the most common claims were related to the composition of the product (e.g., nutrients, ingredients, health-related). Importantly, whenever the products included claims, the sugar content was lower. These results are in line with those found by Angelino et al. [34], namely that breakfast cereals carrying nutrition claims have lower sugar content (vs. without such claims). We also found that products containing other types of claims (e.g., claims related to product origin) had lower sugar content, whereas no differences in sugar content were found according to the presence of hedonic claims. Previous research has shown that nutrition and health claims can help consumers to make healthier and well-informed food choices (for a review, see [53]). Considering that, in our study, products with this type of claim were found to have lower sugar content, future studies should analyze the impact of these claims on consumers’ perceptions about the product as well as their purchase intentions or consumption patterns.

Another packaging feature analyzed was the presentation of the nutritional traffic light. Interestingly, we found several presentation formats that varied on the number of indicators presented or the colors displayed (e.g., black and white, all green, all blue). Our results revealed that products that do not present the nutrition traffic light at the FoP actually had a lower sugar content. The highest sugar content was found for products containing a simplified version that focused on a single indicator—energy per serving size. Hence, although the nutrition traffic light system may constitute a valuable tool for consumers to make more accurate estimates of the products’ healthiness [54], the particular format should not be overlooked. For instance, it is possible that emphasizing the energy content may lead the consumer only to consider this aspect, which is far from ideal to evaluate the sugar content (and overall healthfulness) of a product. Indeed, some authors discuss whether this single-nutrient approach moves consumers away from the overall diet quality with detrimental effects on food choices, consumption, and health [55]. Moreover, the diversity of nutritional labeling systems in the Portuguese marketplace [56] may also hinder consumers’ interpretation of the nutritional profile of food products. Future studies should experimentally examine the impact of visual cues on the interpretation of nutritional traffic lights (and other labeling systems) and address the hypothesis that, in some conditions, this type of information may even bias consumers’ perceptions.

Finally, we explored differences in sugar content according to the presence of cartoon characters. Alarmingly, we found that when packaging presented (vs. did not) these elements, they had more sugar, replicating previous studies conducted in different countries (e.g., [35,57,58]). Considering that breakfast cereals with these characteristics are essentially oriented towards children and, that in Portugal, children and adolescents are those who consume this type of product the most [5], this result raises even greater concerns. Parents are crucial in shaping children’s eating habits because they decide which foods are available in the household [59]. Therefore, it is critical to develop strategies that promote parental awareness that products marketed to children are not necessarily adequate and facilitate healthier food choices.

Overall, as in other countries [8,14,15], the sugar content of most of the breakfast cereals available in Portugal is very high. Our data refer to total sugars (i.e., all mono- and disaccharides regardless of source [3]). It is not possible to distinguish between total and free sugars from the nutritional information presented on the package. Still, breakfast cereals are assumed to have minimal amounts of naturally occurring sugars [60], being mainly produced from grains, which are primarily composed of starch and have very low sugar content (0 to 3 g/100 g [61]). Thus, it is unlikely that our findings regarding sugar content would diverge considerably.

Our results should be generalized with caution, as we selected our sample of breakfast cereals based on the offerings available in only two retailers. Although these retailers hold dominant positions in the Portuguese food market [36,37], and a large number of products and brands were included, we cannot precisely determine how representative the sample is of the product category. Moreover, we did not report consumption data per product exemplar. Having access to that information would allow establishing its relationship with sugar content. Future studies could also examine consumers’ perceptions and consumption patterns of these products.

## 5. Conclusions

The main contribution of our work is approaching the analysis of sugar content of breakfast cereals not only in the context of other nutrients but also regarding other information sources, namely the categorization of the FoP claims and nutritional labeling systems as well as packaging design features. Overall, the results highlight the need to implement strategies to regulate the amount of sugar present in these products to quantities that comply with the current sugar intake guidelines (e.g., product reformulation by the food industry [62]). On the other hand, it is also essential to empower consumers to identify the sugar content of processed foods, for example, through the effective interpretation of the cues included in food packaging. Adopting a government-endorsed policy on nutrition labeling (instead of the multiple systems currently used in Portugal [56]) could be a relevant tool to facilitate the interpretation of nutritional information and to encourage healthier food choices.

## Figures and Tables

**Table 1:** Nutritional information of breakfast cereals (per 100 g).

Nutrient/100 g	*n*	*Min*	*Max*	*Me*	*M*	*SD*
Energy (Kcal)	287	312	501	393	404.0	37.9
Total Fat (g)	287	0.5	29.3	5.9	8.6	7.1
Saturated Fat (g)	287	0	15.6	1.5	2.4	2.5
Total Carbohydrates (g)	287	38	92	69	69.1	11.3
Sugar (g)	286	0	45.2	20.3	19.9	8.8
Fiber (g)	284	1.7	32	6.5	7.3	4.6
Protein (g)	287	4.4	30	8.9	9.2	2.7
Salt (g)	285	0	2.7	0.4	0.5	0.5

*n*, number of breakfast cereals exemplars that included information about a given nutrient; *Min*, minimum; *Max*, maximum; *M*, mean; *Me*, median; *SD*, standard deviation.

**Table 2:** Number and type of claims included in the packaging of breakfast cereals.

Claim Type	Max Claim	Without Claim	With 1 Claim	With 2 Claims	With + 3 Claims
		*n* (%)	*n* (%)	*n* (%)	*n* (%)
Ingredients	4	173 (59.9%)	87 (30.1%)	20 (6.9%)	9 (3.1%)
Nutrients	5	116 (40.1%)	81 (28.0%)	50 (17.3%)	42 (14.5%)
Free from	8	196 (67.8%)	54 (18.7%)	21 (7.3%)	18 (6.2%)
Health-related	4	225 (77.9%)	47 (16.3%)	13 (4.5%)	4 (1.4%)
Low content	3	257 (88.9%)	23 (8.0%)	8 (2.8%)	1 (0.3%)
Flavor	2	226 (78.2%)	50 (17.3%)	13 (4.5%)	0
Sensorial	2	237 (82.0%)	40 (13.8%)	12 (9.0%)	0
Production	3	215 (74.4%)	43 (14.9%)	26 (9.0%)	5 (1.7%)
Place of origin	2	258 (89.3%)	30 (10.4%)	1 (0.3%)	0
Antiquity	1	247 (85.5%)	42 (14.5%)	0	0
Novelty	1	270 (93.4%)	19 (6.6%)	0	0

The minimum amount of any type of claim was zero.

**Table 3:** Top three ingredients for the sample of breakfast cereals.

	First Ingredient		Second Ingredient		Third Ingredient		*Total*
	*n*	%	*n*	%	*n*	%	*n*
Sugar-related ingredients							
Sugar	0	0.0	114	39.4	52	18.0	*166*
Sucrose	0	0.0	6	2.1	1	0.3	*7*
Sugar: Beet	0	0.0	2	0.7	0	0.0	*2*
Sugar: Cane	0	0.0	5	1.7	5	1.7	*10*
Sugar: Coconut	0	0.0	0	0	1	0.3	*1*
Sugar: Organic	0	0.0	1	0.3	0	0.0	*1*
Honey	3	1.0	5	1.7	17	5.9	*25*
Malt	0	0.0	5	1.7	9	3.1	*14*
Maltodextrin	0	0.0	1	0.3	1	0.3	*2*
Caramel	0	0.0	0	0.0	1	0.3	*1*
Syrups	0	0.0	9	3.1	15	5.2	*24*
Other ingredients							
Cereals ^a^	277	95.8	68	23.5	57	19.7	*402*
Fruits, Nuts, Seeds	5	1.7	24	8.3	46	15.9	*75*
Chocolate ^b^	0	0.0	22	7.6	30	10.4	*52*
Oils	0	0.0	7	2.4	24	8.3	*31*
Salt	0	0.0	4	1.4	17	5.9	*21*
Missing/Other	4	1.4	16	5.5	12	4.2	*27*

^a^ The Cereals category includes ingredients such as wheat, corn, oat, barley, or spelt as well as variations such as oat flakes, wheat flour, and maize semolina. ^b^ The Chocolate category includes all variations, such as milk chocolate, dark chocolate, chocolate powder, and chocolate chips.

## Data Availability

The data presented in this study are available on request from the corresponding author.
